# Selective Vulnerability of the Locus Coeruleus Noradrenergic System and its Role in Modulation of Neuroinflammation, Cognition, and Neurodegeneration

**DOI:** 10.3389/fphar.2022.1030609

**Published:** 2022-11-30

**Authors:** Andrew K. Evans, Erwin Defensor, Mehrdad Shamloo

**Affiliations:** School of Medicine, Stanford University, Stanford, CA, United States

**Keywords:** locus coeruleus, noradrenaline, neuroinflammation, oxidative stress, neurodegeneration, beta-adrenergic, Alzheimer’s disease

## Abstract

Locus coeruleus (LC) noradrenergic (NE) neurons supply the main adrenergic input to the forebrain. NE is a dual modulator of cognition and neuroinflammation. NE neurons of the LC are particularly vulnerable to degeneration both with normal aging and in neurodegenerative disorders. Consequences of this vulnerability can be observed in both cognitive impairment and dysregulation of neuroinflammation. LC NE neurons are pacemaker neurons that are active during waking and arousal and are responsive to stressors in the environment. Chronic overactivation is thought to be a major contributor to the vulnerability of these neurons. Here we review what is known about the mechanisms underlying this neuronal vulnerability and combinations of environmental and genetic factors that contribute to confer risk to these important brainstem neuromodulatory and immunomodulatory neurons. Finally, we discuss proposed and potential interventions that may reduce the overall risk for LC NE neuronal degeneration.

## 1 Introduction

**FIGURE 1:**
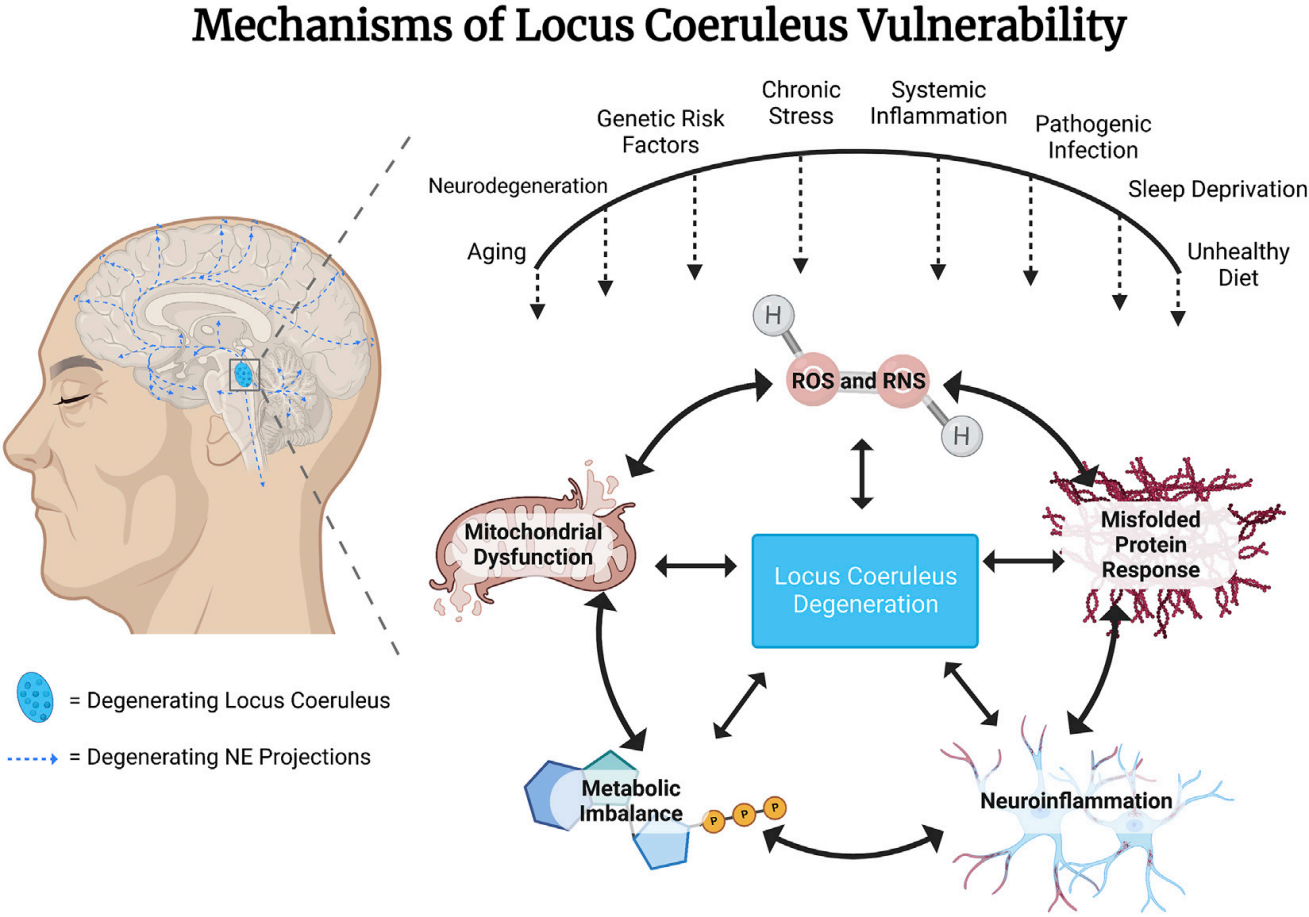
Genetic and environmental risk factors converge to confer vulnerability to locus coeruleus (LC) noradrenergic (NE) neurons. Proximate mechanisms of vulnerability that have been suggested to contribute to LC NE degeneration include actions of reactive oxygen and nitrogen species (ROS/RNS), response to misfolded proteins, neuroinflammatory mechanisms, metabolic imbalance and mitochondrial dysfunction due to high baseline rates of neuronal activity.

Neurons of the locus coeruleus (LC) are the primary source of forebrain norepinephrine (NE). LC NE neurons are activated with waking and have a wide range of firing rates during various behavioral states related to attention, novelty, arousal, and in response to stressors, and their function is essential for adaptation, survival, and multiple aspects of cognitive function (Borodovitsyna et al., 2022; Chandler et al., 2019; Poe et al., 2020; Sara, 2009). The NE system is also essential for the regulation of neuroinflammation (Galea et al., 2003; Heneka et al., 2010; Feinstein et al., 2016; Weinshenker, 2018; Evans et al., 2020). Due to putative mechanisms that we will review here, LC NE neurons are one of the first sites of pathology in the aging brain and are highly susceptible to degeneration (Braak and Del, 2011; Mather and Harley, 2016; Kelly et al., 2017; Weinshenker, 2018). Given the role of the NE system in modulating neuroinflammation, it has been proposed that degeneration of the LC could be a common underlying etiological factor in progressive neuroinflammation observed widely in association with neurodegenerative disorders. An early loss of NE neurons and NE tone may contribute to and accelerate the deterioration of cognitive function and an exacerbation of neuroinflammation, contributing to the worsening of both symptoms and pathology in neurodegenerative disorders. This has opened the possibility that “selective neuronal vulnerability and degeneration” of the LC neurons may be an initiation point for the cascade of pathological events leading to a more widespread pathology and neuronal cell death in the aging and diseased brain (Mather and Harley, 2016). Why do LC neurons display this selective early-onset neuronal vulnerability? What factors determine the aberrant signaling leading to the initiation, temporal profile, and extent of this selective neuronal cell loss? Many possible scenarios have been suggested (see Figure 1), including chronic high metabolic demand, sensitivity to systemic inflammation, viral infection, or toxins in the environment, the persistent activity of oxidizing enzymes such as monoamine oxidase, mitochondrial dysfunction, oxidative and nitrosative stress, and cellular stress in response to misfolded proteins (Kang et al., 2020; Kang et al., 2022) (Zhang et al., 2014a; Wang et al., 2020). Here we review environmental and genetic factors contributing to the vulnerability of the LC and discuss common mechanisms and possible therapeutic interventions to attenuate this vulnerability. The aim of this mini-review is to increase awareness of the multiple risk factors that contribute to the vulnerability of the LC, to identify some common mechanisms of vulnerability, and to initiate further investigation into potential therapeutic interventions and approaches.

### The LC is essential for cognition, attention, arousal, salience, stress adaptation, and modulation of neuroimmune function.

The LC NE system regulates diverse aspects of cognitive function, including arousal, attention, salience, learning and memory, and pain and stress responses; this role in cognitive function has been reviewed extensively (Aston-Jones et al., 2000; Valentino and Van Bockstaele, 2008; Sara, 2009; Roozendaal and McGaugh, 2011; Poe et al., 2020) (Rohampour et al., 2017; Farahani et al., 2021). The LC is a small nucleus comprising between 30,000 and 60,000 neurons in the human brain (German et al., 1988), and it has long been considered a homogeneous and broadly projecting nucleus with diffuse ascending and descending projections. Historically, functional specificity within the NE system has mainly been attributed to differences in excitatory and inhibitory postsynaptic receptor subtypes, receptor distribution patterns, and patterns of neuronal firing rates. However, anatomical subdivisions within the LC and the existence of some functional subsets of NE neurons with specific projection patterns have been described. For example, hippocampal projecting LC NE neurons were shown to be located in the dorsal core of the LC (Mason and Fibiger, 1979). Our understanding of LC function has further evolved in the last 10 years, in particular, as it relates to connectivity to other brain regions and tonic and phasic regulation of neuronal circuits, (Chandler et al., 2019; Poe et al., 2020). Recent investigation has demonstrated a robust functional specificity within subtypes of LC NE neurons. (Chandler et al., 2019). There is a functional topography with subpopulations of neurons receiving distinct inputs and having precise projections related to specific functions (Borodovitsyna et al., 2020). The concept of heterogeneity of the LC NE system complements nicely emerging evidence from other monoaminergic brainstem neuromodulatory systems, such as dopaminergic (Lammel et al., 2012; Beier et al., 2015; Watabe-Uchida and Uchida, 2018) and serotonergic (Lowry et al., 2007; Lowry et al., 2008; Hale and Lowry, 2011) systems. This functional heterogeneity within the LC may confer vulnerability to different subsets of LC NE neurons, which supports evidence for heterogeneity of vulnerability of LC neurons to degeneration in Alzheimer’s disease (AD) (German et al., 1992). Given this heterogeneity within the LC, it becomes less useful to talk about LC activation *versus*, for example, activation of hippocampal or prefrontal cortical projecting LC neurons as functionally defined collections of related neurons. The significance of this is illustrated in studies demonstrating that following bout of stress, LC NE neurons innervating the central nucleus of the amygdala become hyperactive and hyperexcitable. In contrast, those projecting to the prefrontal cortex have suppressed activity and excitability (Chandler et al., 2019). In this case, “activation of the LC” depends on which subpopulation of LC NE neurons is engaged in the specific task. This is especially relevant in the context of vulnerability of LC neurons in relation to chronic activation states.

Recent advances in optogenetic and chemogenetic techniques have enabled precise targeting of NE neuronal firing rates and have advanced our understanding of LC function. Chemogenetic selective activation of the LC has been shown to alter anxiety behavior and to induce large scale functional changes in neural connectivity associated with changes in pupil dilation (Zerbi et al., 2019). In a transgenic rat model (TgF344-AD) in which rats express mutant amyloid precursor protein and presenilin-1, rats show early hyperphosphorylation of tau in the LC, loss of hippocampal and cortical NE fibers, impaired reversal learning in the Morris Water Maze, and behavioral deficits were reversed with chemogenetic activation of the LC (Rorabaugh et al., 2017). In a Ts65Dn mouse model of down syndrome, in which LC NE neurons have been shown to degenerate, chemogenetic activation of LC NE neurons reversed deficits in novel object recognition (Fortress et al., 2015), while chemogenetic inhibition of LC NE neurons resulted in impaired novel object recognition and reversal learning and a potentiation of hippocampal microglia activation (Hamlett et al., 2020).

The NE system is also an important endogenous modulator of neuroinflammation and is involved in the recruitment of peripheral immune cells to the brain (Elenkov et al., 2000; Feinstein et al., 2002; Galea et al., 2003; Heneka et al., 2010; Madrigal et al., 2010; Hinojosa et al., 2011; Gyoneva and Traynelis, 2013). The anti-inflammatory effects of NE have been attributed mainly to the activation of beta-adrenergic receptors (ADRB1 and ADRB2) and modulation of both microglia and astrocyte function. Both ADRB1 and ADRB2 adrenergic receptors are expressed in microglia and astrocytes, although ADRB2 receptors are more highly expressed in both cell types (Zhang et al., 2014b). NE and beta-adrenergic agonists, *via* agonism of beta-adrenergic receptors, reduce the expression of proinflammatory cytokines in the brain (Feinstein et al., 2002; Zapater et al., 2012; Weber et al., 2015; Ardestani et al., 2017; Evans et al., 2020). An ADRB2 agonist, salmeterol, inhibits inflammasome activation and induction of tumor necrosis factor alpha (TNFa) and interleukin 1-beta (IL1b) in a lipopolysaccharide model of systemic inflammation (Song et al., 2018a). Another beta-adrenergic agonist, mabuterol, potentiates IL10 and attenuates macrophage inflammatory protein 1-alpha (MIP1a) in a lipopolysaccharide model of inflammation. Conversely, the beta-adrenergic antagonist propanol attenuates IL10 and potentiates MIP1a, demonstrating the bidirectional effects of noradrenergic pharmacology on the regulation of neuroimmune homeostasis in the brain (Evans et al., 2020). Given this role in the modulation of inflammation, degeneration of LC NE neurons and loss of adrenergic tone may result in dysregulation of neuroimmune homeostasis and potentiation of pathological neuroinflammation in neurodegenerative disorders (Feinstein et al., 2016) and may potentiate both inflammation and neuronal loss in neurodegenerative disorders such as Parkinson’s disease (PD) and AD (Weinshenker, 2008; Heneka et al., 2010; Weinshenker, 2018). Furthermore, the clinical use of beta-blockers for hypertension can further dysregulate the neuroimmune homeostasis in the vulnerable aging population and initiate a cascade of events leading to cognitive decline.

## 2 Pathophysiology of the LC in aging and neurodegenerative disorders – environmental and genetic contributions to vulnerability

Loss of LC NE neurons occurs with normal aging and is accelerated in neurodegenerative disorders such as AD and PD, suggesting these neurons are fundamentally vulnerable to degeneration (Braak and Del, 2011; Kelly et al., 2017; Liu et al., 2019). The LC is one of the first brain regions to develop tau pathology in AD (Braak and Del, 2011) and to show significant early signs of neurodegeneration (German et al., 1992). Early-onset LC degeneration has been reported in patients with mild cognitive impairment before the full-blown manifestation of AD symptoms (Kelly et al., 2017). It has been suggested that degeneration of the LC and the extended LC network may actually precede and be a risk factor for more widespread degeneration in AD patients (Ross et al., 2015; Mather and Harley, 2016; Weinshenker, 2018; Jacobs et al., 2021). Under experimental conditions, loss of NE tone resulting from pharmacological antagonism or lesion of LC NE neurons exacerbates the behavioral deficits, neuroinflammation, and pathology observed in animal models of AD (Heneka et al., 2006; Kalinin et al., 2007; Heneka et al., 2010; Evans et al., 2020). In humans, pathology in the LC also precedes more widespread pathological cell death in neurodegenerative disorders such as AD and PD (Braak and Del, 2011; Betts et al., 2019). In an experimental model of AD, transfection of rat LC neurons with the gene for human mutant tau leads to spreading of pathological tau and behavioral changes related to odor discrimination learning in rats (Ghosh et al., 2019). Consequences of age-related degeneration of the LC NE system may be further exacerbated by the clinical use of brain-permeable beta-adrenergic antagonists, commonly prescribed beta-blockers for controlling hypertension and anxiety, which have been shown to impair cognitive function and potentiate inflammation (Gliebus and Lippa, 2007; Paran et al., 2010; Evans et al., 2020). Epidemiological studies have identified beta-blocker use as a risk factor for chronic neurodegenerative disorders such as PD and AD (Mittal et al., 2017; Cepeda et al., 2019).

Degeneration of the LC can be readily observed and quantified using imaging techniques such as structural MRI, but can also be detected early through more indirect measurement of physiological responses known to be controlled by the LC, such as pupillary dilation reflex, as well as in more complex indicators such as attention, and arousal (Marien et al., 2004; Grudzien et al., 2007; Jacobs et al., 2021; Mather and Harley, 2016; Weinshenker, 2018; Liu et al., 2019; Liu et al., 2020; Matchett et al., 2021). Notably, altered adrenergic signaling with an impact on cognitive function may precede the loss of noradrenergic neurons as the LC becomes dysregulated prior to degeneration (Goodman et al., 2021). This dysregulation may contribute to and precede degeneration as hyperactivity can lead to vulnerability factors such as metabolic and oxidative stress. Why is the LC so susceptible to dysregulation and degeneration? Both environmental and genetic factors may contribute to this vulnerability of LC NE neurons. Environmental risk factors for the development of AD coincide with factors that have been implicated in LC vulnerability, such as chronic stress, poor sleep hygiene, diet, and chronic inflammation (Bredesen, 2015; Ross et al., 2018; Zamore and Veasey, 2022). In terms of biological risk factors, functional studies have identified AD risk-related genes (e.g., TREM2, CD33) linked to the neuroimmune system (Griciuc and Tanzi, 2021) and proteomic changes implicating an altered metabolome (Johnson et al., 2022).

It is important to note that degeneration within the LC is not uniform (German et al., 1992), and biological features of subsets of neurons may contribute to differential vulnerability. Functional subsets of NE neurons, as defined by efferent projections, share morphological features, suggesting fundamental differences in subpopulations of LC NE neurons within the nucleus. New developments in targeting and identifying subpopulations of NE neurons in the LC using genetic, developmental, anatomical, and neurophysiological evidence have further characterized functional subsets of LC neurons with targeted projection sites and involved in specific cognitive functions (Totah et al., 2018; Chandler et al., 2019). Characterizing features of these neuronal subpopulations may help elucidate new mechanisms contributing to LC vulnerability.

### 2.1 Environmental contributors to vulnerability of LC neurons – Inflammation, sleep, diet, stress

Environmental factors that have been shown to impact LC vulnerability include systemic inflammation (Song et al., 2018b; Wang et al., 2020), viral infection, sleep (Zhang et al., 2014a; Zhu et al., 2015; Zamore and Veasey, 2022), diet (Chou et al., 2022) and stress (Ross et al., 2015) (see Figure 1). Many of these environmental factors have been modeled in rodents, enabling mechanistic studies of neurodegeneration and, specifically, LC degeneration. Underlying mechanistic factors contributing to neuronal vulnerability across these models include both oxidative and nitrosative stress, as discussed below.

#### 2.1.1 Systemic inflammation

Systemic inflammation can lead to neuroinflammation (Barrientos et al., 2005; Perry, 2010; Perry and Holmes, 2014) and presents a risk factor for the degeneration of LC NE neurons. Systemic inflammation induced by lipopolysaccharide in animal models has been shown to lead to progressive neurodegeneration with loss of neurons first seen in the locus coeruleus. Neuroinflammation and LC degeneration in this model were reduced in mice with the NADPH oxidase-2 (NOX2) gene deletion or mice treated with a NOX2 inhibitor, implicating NOX2 and production of reactive oxygen and nitrogen species in LC degeneration induced by systemic inflammation (Wang et al., 2020). Systemic inflammation in the presence of primed microglia in the context of aging or chronic neurodegenerative disease triggers a cascade of inflammatory signaling in the brain involving IL1b, neutrophil infiltration, and inducible nitric oxide synthase expression and is associated with increased neuronal death (Barrientos et al., 2005; Cunningham et al., 2005; Cunningham et al., 2009). In patients with Alzheimer’s disease, bouts of systemic inflammation and concentrations of circulating plasma TNFa is predictive of rates of cognitive decline (Holmes et al., 2009). We have observed that a subpopulation of LC NE neurons that are activated in response to systemic inflammation, labeled and chronically tracked with the FosTrap technique, are more susceptible to degeneration over time relative to those LC neurons not activated by systemic inflammation.

#### 2.1.2 Viral infection

Viruses are capable of triggering inflammatory processes in the CNS by direct infiltration or by systemic activation of the immune system (Dahm et al., 2016; Agner and Klein, 2018; Xie et al., 2021). Neurological symptoms of viral infection may include fever, vomiting, chronic fatigue, impaired consciousness, and cognitive deficits (Wasay et al., 2008; Bathoorn et al., 2011; Chen et al., 2021; Wouk et al., 2021). While the acute immune response can be beneficial in promoting CNS repair and slowing down viral replication, chronic and/or irreversible complications that may persist long after viral clearance could be detrimental to brain function (Berth et al., 2009; Amor et al., 2014; Walker, 2018). The LC appears to be particularly vulnerable to toxins and infections, possibly due to extensive innervation of CNS vasculature and close proximity to the fourth ventricle, exposing it to potential pathogens in circulating blood and cerebrospinal fluid (Pamphlett, 2014; Mather and Harley, 2016). Although the causal link between viral infections and neurodegeneration is undetermined, many studies have investigated the association between the two factors and provided hypotheses describing how different viruses may impact neurodegeneration. For instance, herpes simplex virus 1 (HSV-1), which is widely implicated in neurodegenerative disorders (Itzhaki, 2014; Harris and Harris, 2015; Tzeng et al., 2018), can enter a dormant state in the trigeminal ganglion, which projects to the LC (Baringer and Swoveland, 1973). When reactivated, it has been hypothesized that HSV-1 may spread to the LC (Mather and Harley, 2016), where it can induce Aβ accumulation which can contribute to neurodegeneration (Santana et al., 2012; Gupta and Goyal, 2016). The H5N1 influenza virus has also been implicated in neurodegenerative disease. H5N1 infection in a mouse model induced parkinsonian-like symptoms and activated microglia. Alpha-synuclein aggregation, a hallmark of neurodegenerative disorders, was also induced and persisted after the infection was resolved. Behavioral impairments and brain dysfunction have been reported with infection by Severe Acute Respiratory Syndrome Coronavirus 2 (SARS-CoV-2) (Niazkar et al., 2020), the virus that causes COVID-19, with some patients experiencing post-COVID symptoms that may last for years. Symptoms may be related to elevated proinflammatory cytokines, prolonged neuroinflammation, and potentially hypoxia (de Erausquin et al., 2021). The direct viral infection of the brain has also been proposed as a cause for these symptoms, as SARS-CoV-2 viral proteins were detected in the brain tissue of patients who died from COVID-19 (Matschke et al., 2020). It has been suggested that increased COVID-19 susceptibility and severity could increase the risk for neurodegenerative disorders (Li et al., 2022). While further investigations are required to clarify a causal link, these collective findings demonstrate the importance of understanding the neurodegenerative sequelae of viral infections.

#### 2.1.3 Sleep

Sleep deprivation or poor sleep hygiene (e.g., frequent waking, apnea) is a risk factor for Alzheimer’s disease (Ross et al., 2015; Bredesen et al., 2016; Feinstein et al., 2016) and in animal models has been shown to be causally linked to degeneration of LC NE neurons (Zhu et al., 2016). LC NE neurons are most active during wakefulness, with enhanced activity during times of novelty and unpredictability, and are quiet during sleep. LC NE neurons are vulnerable to neurodegeneration following prolonged wakefulness (Zamore and Veasey, 2022). Increased metabolic demand from short bouts of sleep deprivation (i.e., NE neurons remaining active for extended periods of wakefulness) is accompanied by a neuroprotective upregulation of mitochondrial sirtuin 3 (SirT3) signaling (Zhang et al., 2014a). SirT3 has been proposed to have many neuroprotective functions, such as deacetylation of mitochondrial ETC proteins and upregulation of molecules such as glutathione dehydrogenase and activation of isocitrate dehydrogenase, both of which increase levels of reduced glutathione, serving as an important antioxidant. It has been proposed that the extended periods of increased wakefulness and chronic activation of LC NE neurons may also lead to prolonging monoamine production in LC, leading to oxidative stress and shutting off the transient neuroprotective upregulation of mitochondrial SirT3 signaling (Zhang et al., 2014a). Other mechanisms linking extended wakefulness with neuronal vulnerability include NOS activation, mitochondrial stress, and activation of cleaved caspase-3 (Zhang et al., 2014a).

#### 2.1.4 Diet

Insulin resistance and neuroinflammation resulting from a high-fat, high-carbohydrate, Western-style diet have both been identified as risk factors for the development of Alzheimer’s disease (Liu et al., 2011; Bredesen, 2015). Postmortem brain from patients with AD show deficits in the insulin–PI3K–AKT signaling pathway, which could contribute to tau hyperphosphorylation in the LC through activation of glycogen synthase kinase-3β (Liu et al., 2011). Rats sustained on a high-fat carbohydrate-rich diet for 30 weeks show loss of NE neurons in the LC and the substantia nigra (as measured by tyrosine hydroxylase-positive neurons). This cell loss is associated with a deficit in astrocytes (Chou et al., 2022). Astrocytes contribute to a neuroprotective environment by preventing nitrosative damage, as discussed below (Madrigal et al., 2007; Madrigal et al., 2017).

#### 2.1.5 Stress

Stress and elevated glucocorticoid levels engage LC NE neurons (Curtis et al., 2002; Valentino and Van Bockstaele, 2008) and may cause long-term aberrant signaling changes leading to neuronal vulnerability (Ross et al., 2015; Borodovitsyna et al., 2018). Chronic social stress increases mu opioid receptor expression in the LC and results in chronic *suppression* of LC activity that resembles opiate dependence, an effect that endures long after the stress has ended (Chaijale et al., 2013). In light of recent work identifying differential effects of stress on subpopulations of LC NE neurons, this opioid-dependent suppression of LC activity could be within a subpopulation of functionally relevant NE neurons, but this remains to be seen (Borodovitsyna et al., 2020). These changes in response to increased endogenous opioid tone have been linked to corticotrophin-releasing hormone signaling from the amygdala. Social stress also upregulates proinflammatory gene expression in the LC, including IL1β (Wood et al., 2015). Stress increases nitric oxide synthesis in the brain, which may lead to degeneration (Madrigal et al., 2006). In experimental rodent models of stress, chronic unpredictable mild stress results in the generation of reactive oxygen species in the brain coupled with neuroinflammation. The effects of stress on neuroinflammation can be exacerbated in transgenic mice in which the mitochondrial uncoupling protein 2 (UCP2) has been knocked out, as reviewed by (Anderson and Maes, 2014). In humans, psychological stress is associated with increased markers of oxidative stress in students (Sivonova et al., 2004).

### 2.2 Genetic contributors to vulnerability of LC neurons

Genome Wide Association Studies and large-scale proteomic analyses have identified genetic risk factors and biological pathways related to susceptibility to developing AD, several of which may contribute directly to neuronal vulnerability (Zhang et al., 2013; Griciuc and Tanzi, 2021; Johnson et al., 2022). Proteomic analyses have identified biological pathways underlying AD risks, such as cellular metabolism and mitochondrial oxidative stress, as a clear source of vulnerability for the development of AD (Johnson et al., 2022). Genes and proteins that have been identified include Triggering receptors expressed on myeloid cells 2 (TREM2), sialic acid binding Ig-like lectin 3 (Siglec-3; CD33), apolipoprotein E version 4 (APOE4), and mitogen-activated protein kinase (MAPK) signaling (Johnson et al., 2022). Other proteins such as glutamate pyruvate transaminase 2 (GPT2), a metabolic enzyme related to cellular metabolism and the Krebs cycle, implicate mitochondrial metabolism in LC NE neuronal health as KO of *gpt2* in mice results in LC degeneration and are also linked to a neurological developmental disorder in children (Baytas et al., 2022). CD33 mutation impairs phagocytosis of beta-amyloid by microglia (Griciuc et al., 2013; Griciuc et al., 2020). In a newly developed mouse model of genetic risk for sporadic or late-onset Alzheimer’s disease (LOAD mice) transgenic mice express humanized *APOE4* and *TREM2* (Oblak et al., 2021; Oblak et al., 2022) alongside a humanized beta-amyloid precursor protein. This is an important advancement in modeling disease mechanisms in the laboratory as, up until this point, transgenic models have focused mainly on genes related to early onset AD, such as APP and presenilin mutations, which represent only a small fraction of human AD patients. Broadening these models to include genetic risk factors for sporadic or late-onset AD may lead to more scaleable results. Studies combining environmental risk factors with LOAD genetic susceptibility mark a new path forward (Kotredes et al., 2021; Oblak et al., 2022).

### 2.3 Common mechanisms underlying vulnerability of LC neurons

NE neurons of the LC are pacemaker neurons with a high metabolic rate during waking, arousal, and stress. The increased metabolic demand on LC NE neurons, with the extended activity of monoamine oxidase and related accumulation of free oxygen radicals, may contribute to their vulnerability (Zhang et al., 2014a; Wang et al., 2020). Overactivation of NE neurons may also lead to the accumulation of neurotoxic metabolites shown to induce tau pathology (Kang et al., 2020; Kang et al., 2022). NE neurons produce a toxic metabolite, DOPEGAL, when overactivated, which contributes to tau hyperphosphorylation (Kang et al., 2022). Hyperactivation of LC NE neurons may also contribute to mitochondrial oxidative stress through activity-dependent increases in nitric oxide production (Sanchez-Padilla et al., 2014), triggering aberrant cellular signaling and leading to neuronal cell death. LC neurons are also susceptible to tau hyperphosphorylation through alpha-2a adrenergic receptor signaling on LC neurons which, through an autoreceptor mechanism, has been shown to potentiate tau hyperphosphorylation (Zhang et al., 2020). Conversely, antagonism of alpha-2a adrenergic receptors, which blocks autoreceptors on LC neurons and increases NE release, reduces NOS expression and prevents beta-amyloid-induced nitrosative damage (Kalinin et al., 2006). Even under normal physiological conditions, LC NE neurons show higher levels of mitochondrial oxidative stress, which is then exacerbated with further cellular stress inducers such as systemic inflammation (Wang et al., 2020). Aged mice have evidence of oxidative stress and changes in key mitochondrial proteins involved in energy homeostasis in the LC at an early stage before any evident sign of neuronal degeneration (Evans et al., 2021).

Molecular damage attributed to reactive oxygen species (ROS) is common in postmortem brains of Alzheimer’s disease patients, including lipid oxidation and oxidative DNA damage. Beta-amyloid aggregation induces the production of ROS and may mediate some of the cellular damage associated with plaque pathology (Ono et al., 2006). Damage to membrane-bound transporters and ion channels through oxidation has been proposed to underlie compromised mitochondrial function, excitotoxicity, apoptosis, and synaptic degeneration (Mattson, 2004). Beta-amyloid induces astrocytic activation of NADPH oxidase and generation of reactive oxygen species leading to failure of mitochondrial respiration and neuronal death (Abramov et al., 2004). Intracellular beta-amyloid aggregation is also associated with decreased intracellular concentrations of the important endogenous antioxidant glutathione (Madrigal et al., 2007). Understanding the longitudinal progression of the cellular injury and mechanisms underlying selective neuronal vulnerability in the LC is essential for understanding AD pathophysiology and will advance the development of therapeutic interventions.

### 2.4 Therapeutic strategies for protecting LC neurons from oxidative stress and neurodegeneration

**FIGURE 2:**
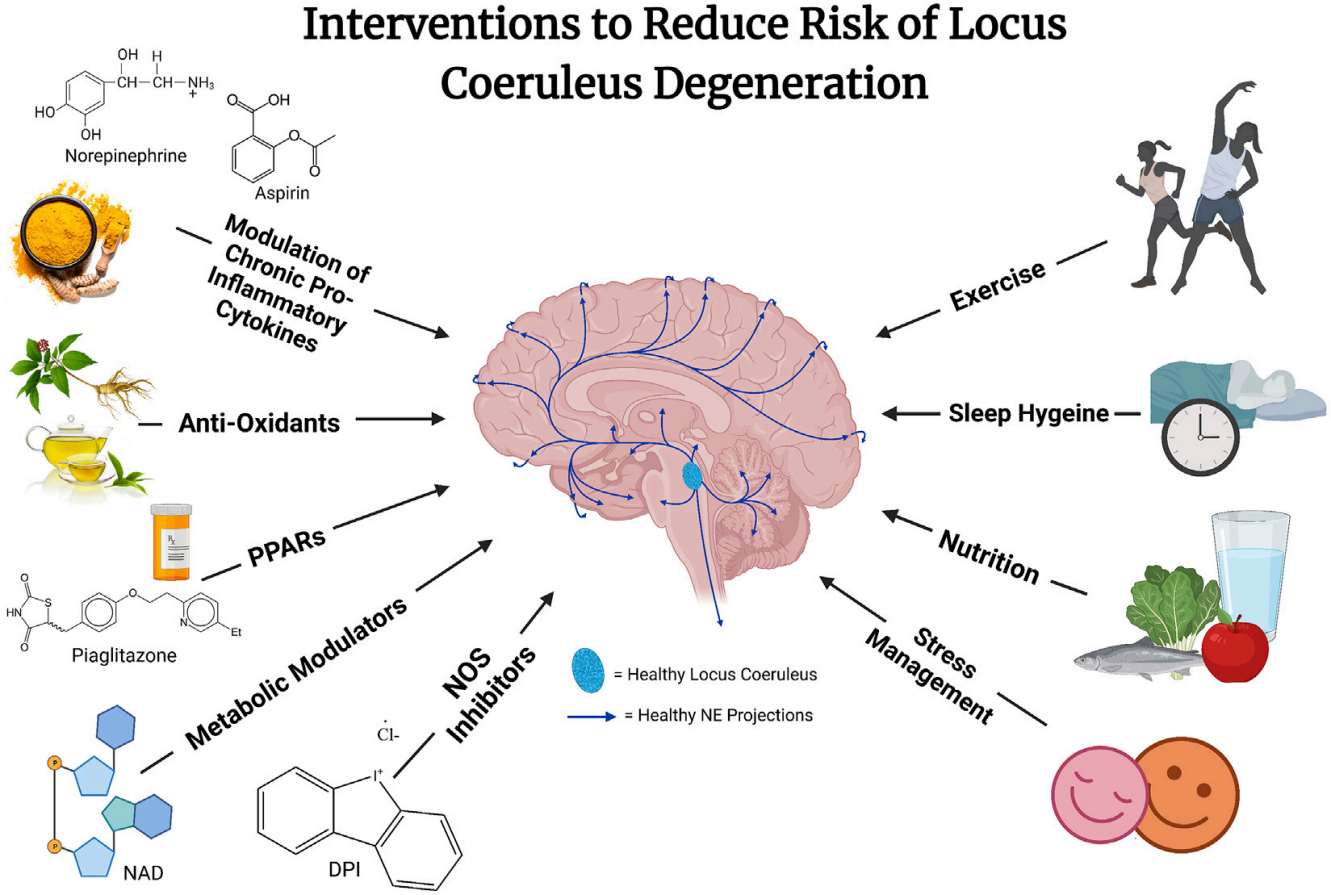
The risk of locus coeruleus degeneration may be reduced through a combination of different interventions. These interventions remain hypothetical and likely will work in concert to reduce vulnerability to what is likely a multi-dimensional disease etiology. Future well designed and controlled studies are needed to explore the efficacy of these approaches.

Multiple strategies combining lifestyle changes such as improved sleep, diet, stress reduction, and physical activity with natural pharmacological therapeutics targeting mechanisms (e.g., reactive oxygen and nitrogen species, inflammation, autophagy, NAD deficiencies, mitochondrial stress) may be effective at preventing or slowing the course of LC or neuronal degeneration (see Figure 2). These hypotheses, while still needing much further validation and confirmation, have been built on preclinical animal studies and epidemiological studies in humans.

#### 2.4.1 Lifestyle factors

Fasting and dietary restriction have been shown to reduce cellular stress and may be neuroprotective (Mattson et al., 2004). Whereas an unhealthy diet may contribute to systemic inflammation and neuroinflammation, healthy nutrition can contribute to a healthy and responsive immune system that can help control systemic inflammation. Balanced nutrition and access to vitamins are essential for a healthy immune system and for replenishing precursors for important biochemical metabolic pathways and antioxidants. Exercise has been shown to have long-term anti-inflammatory effects (De Miguel et al., 2021), and exercise/environmental enrichment is protective against beta-amyloid-induced hippocampal impairment and inflammation in mice (Li et al., 2013; Xu et al., 2018). Whereas short-term effects of exercise have been shown to activate the immune system, this is thought to “train” the immune system and lead to the development of a more responsive immune system capable of being controlled more tightly and reducing the risk of chronic inflammation. Good sleep hygiene allows NE neurons to replenish antioxidant glutathione levels and protective sirtuin pathways, as described above (Zamore and Veasey, 2022). Finally, stress reduction, through a multitude of practices including but not limited to practices such as therapy sessions, meditation, and establishing a strong social support system, has been shown to reduce systemic markers of inflammation, increase cognitive performance, and is thought to reduce risk for psychiatric disease, including neurodegenerative disorders. It should also be noted that these lifestyle factors are also highly interrelated and can be difficult to dissect as exercise is a form of stress reduction, and stress reduction is linked with improved sleep and improved nutrition. The idea that the prevention of neurodegenerative disease may require a multifactorial approach using combined therapies, including lifestyle factors, is gaining momentum in the field (Bredesen et al., 2016).

#### 2.4.2 Modulation of inflammation and oxidative stress

Anti-inflammatory agents, such as non-steroidal anti-inflammatory drugs, have been proposed to reduce the risk for neurodegenerative diseases such as AD, and epidemiological studies have shown mixed results. In mouse models of AD, increasing adrenergic tone results in cognitive improvement and attenuation of beta-amyloid load, neuroinflammation, and tau pathology (Heneka et al., 2010; Coutellier et al., 2014; Ardestani et al., 2017). Chronic inhibition of NOS with a NOS inhibitor, Diphenyleneiodonium (DPI), can be neuroprotective and reverse mitochondrial stress and oxidative damage in animal models of LC degeneration induced by systemic inflammation (Wang et al., 2020). NE inhibits nitric oxide synthase by neuronal production of the neuroprotective CX3CL1 (Feinstein et al., 1993), which reduces nitric oxide production in microglia (Madrigal et al., 2017). NE has been shown to increase the production of the endogenous antioxidant glutathione in a cascade thought to involve the activation of peroxisome proliferator-activated receptors (PPARs) (Madrigal et al., 2007). NE has been suggested to be neuroprotective through its effects on neuroplasticity, inflammation, energy metabolism, and oxidative stress, for review, see (Marien et al., 2004). Antioxidant properties of NE may underlie the neuroprotective effects of NE on dopaminergic neurons in culture (Troadec et al., 2001). A study including four million Norwegians showed a reduced risk for neurodegenerative disease using the commonly prescribed beta-adrenergic agonist, salbutamol. In contrast, the use of the beta-adrenergic antagonist propranolol correlated with increased risk and worsening clinical outcomes (Mittal et al., 2017). This was later confirmed in a follow study by Janssen Pharmaceutical on 117 million people demonstrating adverse effects of the commonly prescribed beta-blocker propranolol (Cepeda et al., 2019).

#### 2.4.3 mTOR, autophagy, and cellular metabolism

mTOR (mechanistic target of rapamycin) is an important serine/threonine protein kinase with a critical regulatory function in cellular and neuronal homeostasis in the regulation of mitochondrial function, energy metabolism, and autophagy. mTOR plays a key pathological role in blocking insulin receptor signaling, autophagy, inflammation, and removal of the misfolded protein aggregates such beta-amyloid. Inhibition of the mTOR has been shown to be neuroprotective in neurodegenerative disorders, as reviewed (Querfurth and Lee, 2021). It has also been suggested and shown that mTOR inhibition can lead to the prolongation of life in mice. This has now been investigated in multiple ongoing human studies (Harrison et al., 2009). Finally, NAD depletion has been suggested to be one of the major factors associated with neurocognitive disorders and associated with neuronal cell death, as reviewed (Guerreiro et al., 2020). Therefore a systemic supplementation of NAD or pharmacological inhibition of the CD38 enzyme responsible for the degradation of the NAD has been explored as an additional potential therapeutic approach (Guerreiro et al., 2020).

## 3 Concluding remarks and future direction

Locus coeruleus noradrenergic neurons are one of the first sites of pathology in neurodegenerative disorders. Their early degeneration, coupled with a loss of noradrenergic tone, can contribute to both cognitive impairment and enhanced pathology and unchecked neuroinflammation associated with these disorders. Risk factors associated with a higher incidence of brain disorders, such as systemic inflammation, have been shown to lead to regional neuroinflammation and chronic degeneration of locus coeruleus noradrenergic neurons in experimental models. These findings warrant further investigation to understand why LC neurons display this selective early-onset neuronal vulnerability in aging and neurodegenerative diseases. What factors determine the aberrant cellular cascades leading to the initiation, temporal profile, and extent of this selective neuronal cell loss? In addition, experimental and epidemiological findings have identified the long-term use of CNS active blood-brain barrier permeable beta-blockers as a risk factor for the deterioration of cognitive function in neurodegenerative disorders such as Parkinson’s and Alzheimer’s. While beta-blockers are important for cardiovascular and blood pressure maintenance, a risk-to-benefit assessment and alternative treatment with a different mode of action should be explored. Our future effort should be focused on the elucidation of mechanistic pathways triggered by risk factors such as systemic inflammation, sleep disruption, chronic stress, and viral infections leading to selective neuronal cell loss in LC and other key brain regions. Identifying underlying mechanisms responsible for the particular vulnerability of LC and examining factors contributing to its resiliency through which protection or prevention may be possible will be essential for understanding the pathophysiology of neurodegenerative disorders and the development of effective therapeutic approaches for its prevention.
